# Cumaceans (Crustacea, Peracarida) associated with shallow-water hydrothermal vents at Banderas Bay, Mexico

**DOI:** 10.3897/BDJ.12.e139801

**Published:** 2024-12-11

**Authors:** María C. Rodríguez-Uribe, Jani Jarquín-González, Patricia Salazar-Silva, Rosa M. Chávez-Dagostino, Natalia Balzaretti Merino

**Affiliations:** 1 Departamento de Ciencias Exactas, Centro Universitario de la Costa, Universidad de Guadalajara, Av. Universidad de Guadalajara 203, CP 48280, Puerto Vallarta, Jalisco, Mexico Departamento de Ciencias Exactas, Centro Universitario de la Costa, Universidad de Guadalajara, Av. Universidad de Guadalajara 203, CP 48280 Puerto Vallarta, Jalisco Mexico; 2 División de Estudios de Posgrado e Investigación, Instituto Tecnológico de Chetumal, Tecnológico Nacional de México, Av. Insurgentes 330, CP 77013, Chetumal, Quintana Roo, Mexico División de Estudios de Posgrado e Investigación, Instituto Tecnológico de Chetumal, Tecnológico Nacional de México, Av. Insurgentes 330, CP 77013 Chetumal, Quintana Roo Mexico; 3 Departamento de Ingenierías, Tecnológico Nacional de México, Instituto Tecnológico de Bahía de Banderas, crucero a Punta Mita S/N, CP 63734, La Cruz de Huanacaxtle, Nayarit, Mexico Departamento de Ingenierías, Tecnológico Nacional de México, Instituto Tecnológico de Bahía de Banderas, crucero a Punta Mita S/N, CP 63734 La Cruz de Huanacaxtle, Nayarit Mexico; 4 Departamento de Ciencias Biológicas, Centro Universitario de la Costa, Universidad de Guadalajara, Av. Universidad de Guadalajara 203, CP 48280, Puerto Vallarta, Jalisco, Mexico Departamento de Ciencias Biológicas, Centro Universitario de la Costa, Universidad de Guadalajara, Av. Universidad de Guadalajara 203, CP 48280 Puerto Vallarta, Jalisco Mexico; 5 Departamento de Artes, Educación y Humanidades, Centro Universitario de la Costa, Universidad de Guadalajara, Av. Universidad de Guadalajara 203, CP 48280, Puerto Vallarta, Jalisco, Mexico Departamento de Artes, Educación y Humanidades, Centro Universitario de la Costa, Universidad de Guadalajara, Av. Universidad de Guadalajara 203, CP 48280 Puerto Vallarta, Jalisco Mexico

**Keywords:** benthos, fine sand, infauna, morphospecies, nomenclatural status, pH

## Abstract

**Background:**

Cumaceans mostly inhabit marine environments, where they play a crucial role in marine food webs and actively participate in the transfer between benthic and pelagic systems. Scientific interest in these crustaceans has been increasing, but is limited to certain geographic areas, which do not include extreme environments such as hydrothermal vents.

**New information:**

Therefore, this study aimed to report the distribution of cumaceans in shallow-water hydrothermal vents at Banderas Bay and to identify the specimens present. Three sites were selected (20°44’54.7”N, 105°28’40.6”W; 20°44’54.8”N, 105°28’40.4”W; 20°44’54.9”N, 105°28’38.4”W) and each site was divided into three zones, based on sediment temperature. Through SCUBA diving, 27 sediment cores were collected. The samples were processed and identified in the laboratory. The families Bodotriidae T. Scott, 1901; Pseudocumatidae Sars, 1878; and Diastylidae Bate, 1856; were recorded and six morphospecies were identified. This work leaves a preliminary frame of reference for future studies related to the biodiversity of cumacean in Hydrothermal vents environments.

## Introduction

Cumaceans are small crustaceans of the class Malacostraca and the superorder Peracarida (Corbera 2015). These organisms lack larval stages, are benthic and epibenthic and are generally found in marine habitats, although they have also been reported to be in brackish and freshwater environments (Heard et al. 2007, Jarquín-González and García-Madrigal 2013). They play an important role in the food webs of some benthic communities because they are considered the main food source for juvenile fish, decapods and starfish (Moore et al. 2007). They can be found on sandy, coral and rocky substrates, as well as epibionts of encrusting organisms (e.g. algae, sponges, hydroids, bryozoans) (Heard et al. 2007, Jarquín-González and García-Madrigal 2013, Uhlir et al. 2021) and even associated with whale carcasses and hydrothermal vents (Cristales and Pires-Vanin 2014).

Despite the growing scientific interest in the order Cumacea in recent decades, there remains a general lack of knowledge about these organisms. According to Gerken et al. (2022), this is primarily due to various factors, including the small size of these organisms (1-30 mm), challenges related to the definition and clarification of diagnostic characters at the family level, difficulties in obtaining biological samples in the field, the use of fixation procedures (e.g. formalin) that do not allow for DNA preservation and the fact that molecular sequences have not yet been obtained for some species or that the procedures have not been successful. Consequently, knowledge about cumaceans is limited, specially in geographic regions such as hydrothermal vents (HV). These are recognised as two distinct phenomena: deep-sea (> 200 m) (DSHV) and shallow-water (< 200 m) (SWHV) hydrothermal vents (Tarasov et al. 2005). They have different physical, chemical and biological characteristics, in addition to the depth at which they are found, which clearly differentiates them. Most of these HV are related to volcanic activity, resulting in the release of gases enriched in volcanic volatiles, which generate more acidic fluids and cause the leaching of magnesium (Mg) and other elements, especially metals, from the host rocks (Yang and Scott 1996, Reeves et al. 2011). Furthermore, according to Melwani and Kim (2008), the infauna associated with SWHV tend to display different adaptations such as tolerance and detoxification mechanisms, as they develop and use structures (e.g. tubes, shells) or take advantage of their mobility to move away from the extreme conditions and, thus, be able to survive permanently or temporarily in these environments.

Knowledge about cumaceans present in HV comes mainly from ecological reports on communities or structural components of the meiofauna, both in DSHV (Smith et al. 2002, Desbruyeres et al. 2006, Corbera 2006, Klunder et al. 2020) and SWHV (Kamenev et al. 1993, Tarasov 2006, Zeppilli and Danovaro 2009, Donnarumma et al. 2019, Baldrighi et al. 2020, Taylor et al. 2021, Wang et al. 2022, Rodríguez-Uribe et al. 2023). However, there are also the research articles of Corbera et al. (2008) and Corbera and Segonzac (2010), who contribute to the taxonomy of this group, since they have reported new genera and species associated with DSHV. Currently, no new genera or species have been recognised for SWHV; however, what has been reported before 2010 in these environments are six families, nine genera, 10 species and five morphospecies of cumaceans associated with SWHV (see below).

In the coastal area of the Mexican Pacific, including the Gulf of California, different species and morphospecies of cumaceans have been reported, mainly associated with coral reefs, macroalgae, seagrass beds and sandy beaches with fine sediments. These investigations cover different locations, from San Quintin Bay and Baja California to the coasts of Guerrero, Oaxaca and Chiapas (see below). However, in the Mexican Pacific, the biodiversity of cumaceans associated with SWHV is limited to the study of Melwani and Kim (2008) who reported three families, three genera, one species and three morphospecies for Bahía Concepción, Baja California (see below). While for the SWHV of Punta Mita at Banderas Bay, Nayarit, there is only the work of Rodríguez-Uribe et al. (2023) who documented the presence of cumaceans, but at a supraspecific level (order).

Although the presence of cumaceans in some SWHVs has been reported, there is still a notable lack of scientific effort dedicated to generating knowledge about these organisms in these environments. This research aimed to document the faunal composition of cumaceans associated with the SWHV of Punta Mita, located in Banderas Bay, Mexico, to leave a preliminary frame of reference for future studies related to the biodiversity of the Cumacea order in HV environments.

## Materials and methods

**Figure 1.**
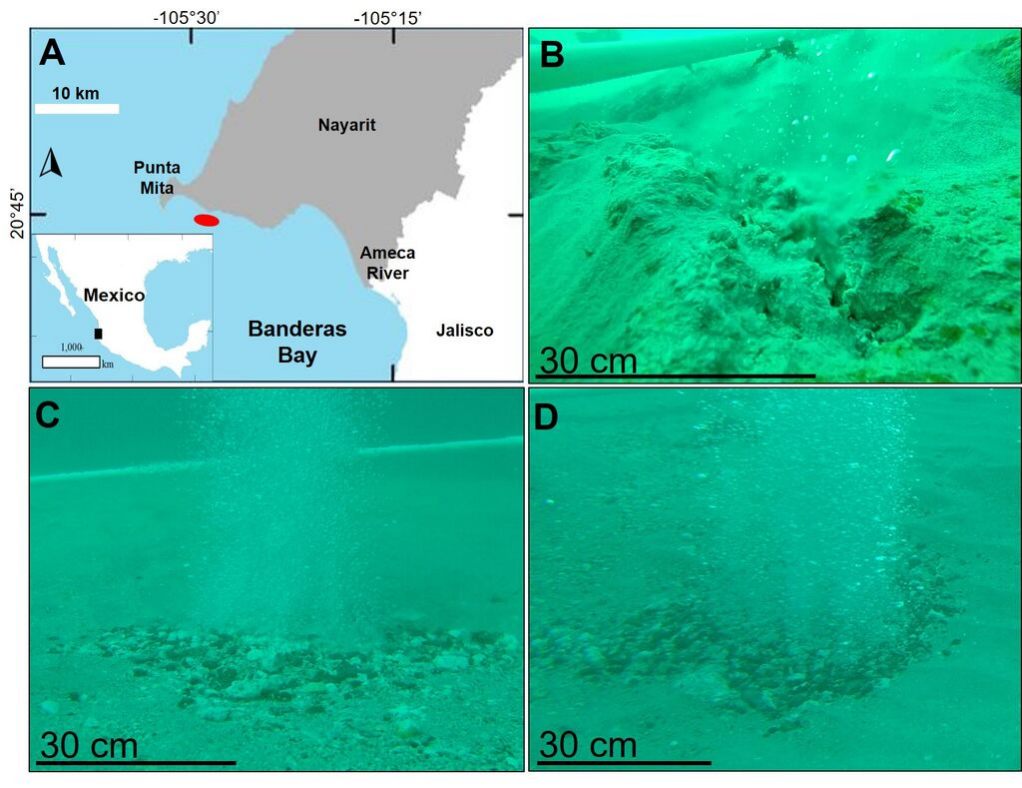
Study area. **A** The red oval indicates the location of the shallow-water hydrothermal vents of Punta Mita. Images **B, C** and **D** show the three hydrothermal vents of each study site, at the time of a hydrothermal discharge. (B) site 1 vent, (C) site 2 vent and (D) site 3 vent.

The SWHV of Punta Mita is located on the northern coast of Banderas Bay, Nayarit, Mexico, 400 m from the beach, 5 km south of Punta Mita and at a depth of 10 m (Fig. 1). This SWHV has an area of influence of approximately 1 km^2^ (Canet and Prol-Ledesma 2006). The hydrothermal discharges are composed of liquid and gases, where the exhaled water is less saline than seawater and enriched in Si, Ca, Li, B, Ba, Fe, Mn and As (Prol-Ledesma et al. 2002), while the gas is mostly composed of N_2_ (88%) and CH_4_ (12%) (Prol-Ledesma et al. 2002). In November 2017, a sampling campaign was carried out at the SWHV of Punta Mita where three sites with hydrothermal activity were selected, which reached temperatures of up to 87.5°C Site 1 (S1) (20°44’54.7”N; 105°28’40.6”W), Site 2 (S2) (20°44’54.8”N; 105°28’40.4”W), and Site 3 (S3) (20°44’54.9”N; 105°28’38.4”W).

Each site was divided into three square zones (n = 9). These divisions were made, based on the bottom temperature and the proximity to the centre of the hydrothermal vent. Zone 1 (Z1) had the hydrothermal vent at its centre, recording the highest temperatures and an area of 0.25 m^2^, zone 2 (Z2) is the zone with intermediate temperature and an area of 9 m^2^ and zone 3 (Z3) with ambient temperature and an area of 36 m^2^. Sediment samples were collected using plastic cores (PVC of 10 x 10 cm and 10 cm in diameter) by SCUBA diving in each zone of each study site, with three samples per zone (n = 27). A YSI™ Professional 1030 multiparameter probe (Pro1030) was used to record the pH, conductivity, salinity and seawater temperature at each study area. The cores with the sediment samples were kept frozen for subsequent processing and were filtered during laboratory work. Each sample was sieved using an 8” ALCON™ brass sieve number 20 with a mesh size of 850 µm and, under an Optika™ 50 stereoscopic microscope (Via Rigla, Bergamo, Italy), the specimens found were separated by site and zone. They were preserved in 96% alcohol for later identification. These samples were stored at ambient temperature in the laboratory.

The identification process was carried out by Dr. Jani Jarquín-González at the laboratory of Ecología Trófica of the Instituto Tecnológico de Chetumal, Quintana Roo, Mexico. To corroborate the diagnostic characteristics, the specimens were mounted on a slide with glycerol and examined under a VELAB™ Stereo VE-S5 microscope. The cumaceans were identified to the highest possible taxonomic level, for which the keys of Watling and McCann (1997), Haye (2007) and Jarquín-González and García-Madrigal (2013) were consulted, as well as the works of Sars (1894), Heard et al. (2007) and Gerken (2016). The species checklist and nomenclatural status were assigned following the editorial advice of WoRMS (2023). Photographs of the representative specimens of each morphotype were taken using a VELAB™ VE-LX1800 digital camera. The biological material is deposited in the Personal Reference Collection of Dr. María C. Rodríguez-Uribe, located in the Hydrothermal Systems office of the Centro Universitario de la Costa, University of Guadalajara, Puerto Vallarta, Mexico.

## Taxon treatments

### Cyclaspis sp. 1

FCCCEC59-960D-5991-B468-F97441BD20BF

#### Materials

- **Type status:** Other material. **Occurrence:** recordedBy: María C. Rodríguez-Uribe, Rosa M. Chávez-Dagostino, Natalia Balzaretti Merino; individualCount: 2; sex: male; lifeStage: adult; occurrenceID: 3913556F-A19A-5CA0-AA23-EE849AB7E96A; **Taxon:** class: Malacostraca; order: Cumacea; family: Bodotriidae; genus: Cyclaspis; taxonRank: genus; **Location:** higherGeography: Mexican Central Pacific; continent: American; waterBody: Banderas Bay; country: Mexico; stateProvince: Nayarit; locality: shallow-water hydrothermal vents of Punta Mita; verbatimDepth: 10 m; verbatimCoordinateSystem: 20°44’54.9”N 105°28’38.4”W; **Event:** year: 2017; month: 11; day: 17; habitat: in fine sand

#### Description

**Figure 2.**
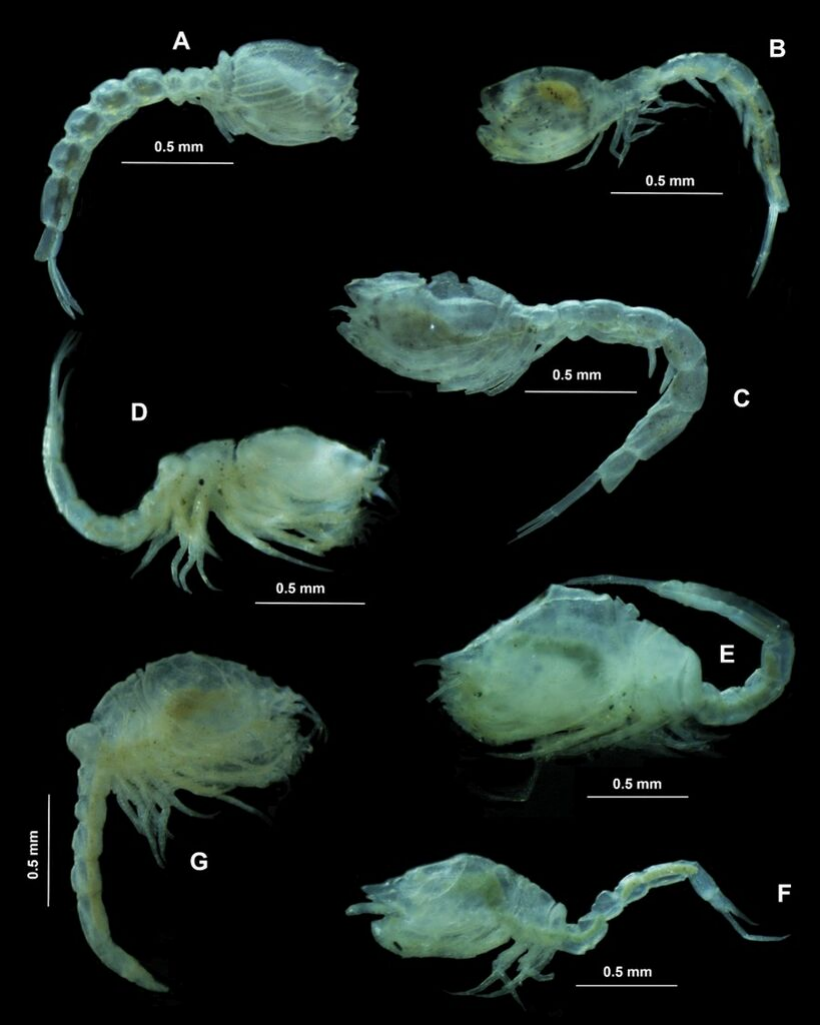
Representative cumacean morphospecies of each family. **A-C** Bodotriidae: *Cyclaspis* sp. 1 (A); *Cyclaspis* sp. 2 (B); *Pseudoleptocuma* sp. 1 (C); **D-F** Diastylidae: *Diastylis* sp. 1 (D); *Oxyurostylis* sp. 1, male (E); *Oxyurostylis* sp. 1, female; **G** Pseudocumatidae: *Pseudocuma* sp. 1 (G).

The carapace is shorter than the abdomen and longer than the pereon; the abdomen is slightly longer than the carapace and pereon together. Carapace with anterior transverse ridges and several mid-posterior ridges; with two teeth on the mid-dorsal line. With short branchial siphons. Antennal notch as a subacute incision. The frontal lobe is about 1/4 of the carapace length. With ocular pigment. Pereonite 2 narrowing to other pereonites. Pereonite 4 overriding pereonite 5. Pereopod 2 with ischium. Pleonites rounded. Pleonite 6 is shorter than the peduncle of the uropod. With five pairs of pereopods. Uropodal peduncle longer than rami. Uropodal endopod uni-articulated (Fig. 2A).

### Cyclaspis sp. 2

43BC0EB8-5C76-5B9A-9D7A-2562B3C63A16

#### Materials

- **Type status:** Other material. **Occurrence:** recordedBy: María C. Rodríguez-Uribe, Rosa M. Chávez-Dagostino, Natalia Balzaretti Merino; individualCount: 5; sex: male; lifeStage: adult; occurrenceID: 4098186B-9266-53C0-9670-5EE5D7E43222; **Taxon:** class: Malacostraca; order: Cumacea; family: Bodotriidae; genus: Cyclaspis; taxonRank: genus; **Location:** higherGeography: Mexican Central Pacific; continent: American; waterBody: Banderas Bay; country: Mexico; stateProvince: Nayarit; locality: shallow-water hydrothermal vents of Punta Mita; verbatimDepth: 10 m; verbatimCoordinateSystem: 20°44’54.8”N 105°28’40.4”W; **Event:** year: 2017; month: 11; day: 17; habitat: in fine sand
- **Type status:** Other material. **Occurrence:** recordedBy: María C. Rodríguez-Uribe, Rosa M. Chávez-Dagostino, Natalia Balzaretti Merino; individualCount: 1; sex: female; lifeStage: adult; reproductiveCondition: ovate; occurrenceID: CC2E23BA-45B9-5104-9659-D19E35F015BE; **Taxon:** class: Malacostraca; order: Cumacea; family: Bodotriidae; genus: Cyclaspis; taxonRank: genus; **Location:** higherGeography: Mexican Central Pacific; continent: American; waterBody: Banderas Bay; country: Mexico; stateProvince: Nayarit; locality: shallow-water hydrothermal vents of Punta Mita; verbatimDepth: 10 m; verbatimCoordinateSystem: 20°44’54.8”N 105°28’40.4”W; **Event:** year: 2017; month: 11; day: 17; habitat: in fine sand
- **Type status:** Other material. **Occurrence:** recordedBy: María C. Rodríguez-Uribe, Rosa M. Chávez-Dagostino, Natalia Balzaretti Merino; individualCount: 3; sex: female; lifeStage: adult; reproductiveCondition: non-ovate; occurrenceID: 335693EB-D314-50E6-A0C9-1240357632EC; **Taxon:** class: Malacostraca; order: Cumacea; family: Bodotriidae; genus: Cyclaspis; taxonRank: genus; **Location:** higherGeography: Mexican Central Pacific; continent: American; waterBody: Banderas Bay; country: Mexico; stateProvince: Nayarit; locality: shallow-water hydrothermal vents of Punta Mita; verbatimDepth: 10 m; verbatimCoordinateSystem: 20°44’54.8”N 105°28’40.4”W; **Event:** year: 2017; month: 11; day: 17; habitat: in fine sand
- **Type status:** Other material. **Occurrence:** recordedBy: María C. Rodríguez-Uribe, Rosa M. Chávez-Dagostino, Natalia Balzaretti Merino; individualCount: 1; sex: female; lifeStage: adult; reproductiveCondition: non-ovate; occurrenceID: F588D5B0-973A-57CB-B1E5-0E88AE7FE9AC; **Taxon:** class: Malacostraca; order: Cumacea; family: Bodotriidae; genus: Cyclaspis; taxonRank: genus; **Location:** higherGeography: Mexican Central Pacific; continent: American; waterBody: Banderas Bay; country: Mexico; stateProvince: Nayarit; locality: shallow-water hydrothermal vents of Punta Mita; verbatimDepth: 10 m; verbatimCoordinateSystem: 20°44’54.9”N 105°28’38.4”W; **Event:** year: 2017; month: 11; day: 17; habitat: in fine sand

#### Description

The carapace is shorter than the abdomen and longer than the pereon; the abdomen is subequal to the carapace and pereon together. Carapace with smooth transversal ridges; with four teeth on the mid-dorsal line (females). With short branchial siphons. Antennal notch as an acute incision. The frontal lobe is about 1/5 of the carapace length. With ocular pigment. Pereonite 2 narrow to other pereonites. Pereonite 2 overriding pereonite 1 and 3. Pereopod 2 with ischium. Pleonites cylindrical. Pleonite 6 is shorter than the peduncle of the uropod. Male with five pairs of pereopods. Peduncle of uropod longer than rami. Uropodal endopod uniarticulated. Uropodal peduncle without spines or setae on inner margin (females), uropodal exopod with four spines on outer margin (females) (Fig. 2B).

### Pseudoleptocuma sp. 1

78F6F38C-4ADC-5A9B-A2B0-214FD15ABAED

#### Materials

- **Type status:** Other material. **Occurrence:** recordedBy: María C. Rodríguez-Uribe, Rosa M. Chávez-Dagostino, Natalia Balzaretti Merino; individualCount: 2; sex: male; lifeStage: adult; occurrenceID: 1108946C-4BFF-5EF8-8FD5-45E519913F8C; **Taxon:** class: Malacostraca; order: Cumacea; family: Bodotriidae; genus: Pseudoleptocuma ; taxonRank: genus; **Location:** higherGeography: Mexican Central Pacific; continent: American; waterBody: Banderas Bay; country: Mexico; stateProvince: Nayarit; locality: shallow-water hydrothermal vents of Punta Mita; verbatimDepth: 10 m; verbatimCoordinateSystem: 20°44’54.7”N 105°28’40.6”W; **Event:** year: 2017; month: 11; day: 17; habitat: in fine sand

#### Description

Carapace shorter than abdomen and longer than pereon; abdomen longer than carapace and pereon together. From a dorsal view, the carapace appears laterally compressed anteriorly. Antennal notch as a subacute incision, with an antero-lateral corner with the acute tooth. With ocular pigment. The first pereonite is visible only above the lateral mid-line. Pereonite 2 with ventrolateral expansion overriding pereonite 1 and carapace. Pereonite 3 extended forwards and backwards overriding pereonites 2 and 4. Pereonite 4 with ventrolateral expansion overriding pereonite 5. Pereopods 1 with exopod. The whole width of the terminal end of pleonite 6 slightly extended between the bases of the uropods; the apex rounded. Uropodal endopod uni-articulated. Uropodal exopod bi-articulated, with proximal article shorter than distal one. Uropodal peduncle longer than rami. Rami is approximately the same length. Males with three pairs of pleopods (Fig. 2C).

### Diastylis sp. 1

D555E838-7C3F-549A-904F-7039EE729540

#### Materials

- **Type status:** Other material. **Occurrence:** recordedBy: María C. Rodríguez-Uribe, Rosa M. Chávez-Dagostino, Natalia Balzaretti Merino; individualCount: 1; sex: male; lifeStage: adult; occurrenceID: CA9DE74D-95FC-5792-B4CA-2A9A43F4E7C1; **Taxon:** class: Malacostraca; order: Cumacea; family: Diastylidae; genus: Diastylis; taxonRank: genus; **Location:** higherGeography: Mexican Central Pacific; continent: American; waterBody: Banderas Bay; country: Mexico; stateProvince: Nayarit; locality: shallow-water hydrothermal vents of Punta Mita; verbatimDepth: 10 m; verbatimCoordinateSystem: 20°44’54.8”N 105°28’40.4”W; **Event:** year: 2017; month: 11; day: 17; habitat: in fine sand

#### Description

The carapace is tumid, with lateral and transversal ridges. The antenna does not extend beyond pereonite 5 (male). Third and fourth pereonites not coalesced. Pereopods 3 and 4 with small exopods. Telson with one pair of subdistal setae. With two pairs of pleopods (male). Telson is longer than the sixth pleonite (considering the post-anal part). The post-anal part of the telson is longer than the pre-anal part. Uropodal endopod tri-articulated (Fig. 2D).

### Oxyurostylis sp. 1

7AD4C4AB-6691-58F1-8743-6EDF6C14D8FF

#### Materials

- **Type status:** Other material. **Occurrence:** recordedBy: María C. Rodríguez-Uribe, Rosa M. Chávez-Dagostino, Natalia Balzaretti Merino; individualCount: 4; sex: male; lifeStage: adult; occurrenceID: 8C766392-B816-5F1B-B700-D0849CBD90E1; **Taxon:** class: Malacostraca; order: Cumacea; family: Diastylidae; genus: Oxyurostylis; taxonRank: genus; **Location:** higherGeography: Mexican Central Pacific; continent: American; waterBody: Banderas Bay; country: Mexico; stateProvince: Nayarit; locality: shallow-water hydrothermal vents of Punta Mita; verbatimDepth: 10 m; verbatimCoordinateSystem: 20°44’54.8”N 105°28’40.4”W; **Event:** year: 2017; month: 11; day: 17; habitat: in fine sand
- **Type status:** Other material. **Occurrence:** recordedBy: María C. Rodríguez-Uribe, Rosa M. Chávez-Dagostino, Natalia Balzaretti Merino; individualCount: 4; sex: female; lifeStage: adult; reproductiveCondition: non-ovate; occurrenceID: 6E689E5C-E5B6-58EA-BDD7-E7F8A5682238; **Taxon:** class: Malacostraca; order: Cumacea; family: Diastylidae; genus: Oxyurostylis; taxonRank: genus; **Location:** higherGeography: Mexican Central Pacific; continent: American; waterBody: Banderas Bay; country: Mexico; stateProvince: Nayarit; locality: shallow-water hydrothermal vents of Punta Mita; verbatimDepth: 10 m; verbatimCoordinateSystem: 20°44’54.8”N 105°28’40.4”W; **Event:** year: 2017; month: 11; day: 17; habitat: in fine sand

#### Description

Carapace with horizontal and oblique ridges; with spines on lateral margins, dorsal surface and rostrum. Pereopods 3 and 4 with small exopods. Telson is not fused to the sixth pleonite. Telson is longer than the sixth pleonite (considering the post-anal part). Telson is long and narrow; without apical setae; terminating in an acute styliform tip (Fig. 2E and F).

### Pseudocuma sp. 1

4111746C-0F16-5481-90EF-39A1085E47B2

#### Materials

- **Type status:** Other material. **Occurrence:** recordedBy: María C. Rodríguez-Uribe, Rosa M. Chávez-Dagostino, Natalia Balzaretti Merino; individualCount: 1; sex: male; lifeStage: adult; occurrenceID: 77AF762B-F05F-561D-A050-33F0C622A362; **Taxon:** class: Malacostraca; order: Cumacea; family: Pseudocumatidae; genus: Pseudocuma; taxonRank: genus; **Location:** higherGeography: Mexican Central Pacific; continent: American; waterBody: Banderas Bay; country: Mexico; stateProvince: Nayarit; locality: shallow-water hydrothermal vents of Punta Mita; verbatimDepth: 10 m; verbatimCoordinateSystem: 20°44’54.8”N 105°28’40.4”W; **Event:** year: 2017; month: 11; day: 17; habitat: in fine sand
- **Type status:** Other material. **Occurrence:** recordedBy: María C. Rodríguez-Uribe, Rosa M. Chávez-Dagostino, Natalia Balzaretti Merino; individualCount: 1; sex: female; lifeStage: adult; reproductiveCondition: non-ovate; occurrenceID: FAEFDB21-8493-5861-810E-4B42A039BBBC; **Taxon:** class: Malacostraca; order: Cumacea; family: Pseudocumatidae; genus: Pseudocuma; taxonRank: genus; **Location:** higherGeography: Mexican Central Pacific; continent: American; waterBody: Banderas Bay; country: Mexico; stateProvince: Nayarit; locality: shallow-water hydrothermal vents of Punta Mita; verbatimDepth: 10 m; verbatimCoordinateSystem: 20°44’54.8”N 105°28’40.4”W; **Event:** year: 2017; month: 11; day: 17; habitat: in fine sand
- **Type status:** Other material. **Occurrence:** recordedBy: María C. Rodríguez-Uribe, Rosa M. Chávez-Dagostino, Natalia Balzaretti Merino; individualCount: 1; sex: male; lifeStage: juvenile; occurrenceID: 3994A9F2-73EB-5587-915F-A59B1E7E0EF6; **Taxon:** class: Malacostraca; order: Cumacea; family: Pseudocumatidae; genus: Pseudocuma; taxonRank: genus; **Location:** higherGeography: Mexican Central Pacific; continent: American; waterBody: Banderas Bay; country: Mexico; stateProvince: Nayarit; locality: shallow-water hydrothermal vents of Punta Mita; verbatimDepth: 10 m; verbatimCoordinateSystem: 20°44’54.8”N 105°28’40.4”W; **Event:** year: 2017; month: 11; day: 17; habitat: in fine sand

#### Description

Body short and compact. Carapace with branchial regions well defined, and the antero-lateral corners more or less produced. Pseudorostrum prominent. Eye well developed. Pereon with 5 well-defined segments. Pereon of the usual slender cylindrical form. Telson is very small and unarmed, but distinctly defined from the last pleonite. With two rudimentary pleopods (male) (Fig. 2G).

## Analysis

**Distribution of cumaceans in the Shallow-water hydrothermal vents of Punta Mita**

A total of 29 cumaceans were collected in the three study sites. In S1, two cumaceans (6.90%) were found, in S2, 22 (75.86%) and in S3, five (17.24%) (Table 1). The largest number of cumaceans was collected in S2, while S1 recorded the lowest. Of these 29 cumaceans, only 26 specimens could be analysed, as three of them were incomplete (e.g. without pereopods or uropods) and had partially destroyed shells, which made their identification impossible; the rest of the specimens presented minor damage, thus allowing their identification at the morphospecies level.

The average physical and chemical parameters recorded at the three study sites were calculated by zones. For the Z1, the values were as follows: pH of 7.678 ± 0.022, conductivity 46.045 ± 0.572 mS/cm, salinity 18.585 ± 1.218 ppt and temperature of 87 ± 0.67°C. In Z2, the values were: pH of 7.988 ± 0.042, conductivity 53.183 ± 1.152 mS/cm, salinity 33.25 ± 1.817 ppt and temperature of 28.32 ± 2.18°C. While in Z3: pH of 8.048 ± 0.008, conductivity 54.317 ± 1.117 mS/cm, salinity 35 ± 0.567 ppt and temperature of 26.88 ± 0.617°C.

**Identification of cumaceans**

Of the 26 specimens analysed, six morphospecies of cumaceans were recognised: *Cyclaspis* sp. 1, *Cyclaspis* sp. 2, *Diastylis* sp. 1, *Oxyurostylis* sp. 1, *Pseudoleptocuma* sp. 1 and *Pseudocuma* sp. 1 (Fig. 2) (Table 1). In addition, of this total of specimens, it was determined that 14 (53.9%) belong to the Bodotriidae T. Scott, 1901 family; nine (34.6%) to the Diastylidae Bate, 1856 family; and three (11.5%) to the Pseudocumatidae Sars, 1878 family.

The families Bodotriidae, Diastylidae and Pseudocumatidae and the genera *Cyclaspis* Sars, 1865, *Diastylis* Say, 1818, *Oxyurostylis* Calman, 1912, *Pseudocuma* G.O. Sars, 1865 and *Pseudoleptocuma* Watling, 1977 are recorded for the study area for the first time. It is noted that both the family Pseudocumatidae and the genus *Pseudocuma* are new records for the Eastern Tropical Pacific, while the genus *Pseudoleptocuma* is recorded for the first time for the Mexican Pacific. Additionally, the names of two species of cumaceans recorded for the Mexican Pacific are updated (Table 2).

## Discussion

The distribution of cumaceans in the SWHV of Punta Mita shows that the Z1s represent the zones with direct hydrothermal influence since they are the closest to the hydrothermal vents. These zones recorded the highest temperatures (87.5°C) and the lowest pH values (7.635), presenting the lowest amounts of cumaceans (n = 0, 6, 1) in the three study sites (Table 1). These results suggest that the extreme conditions of high temperature and low pH in Z1 zones limit the presence of these organisms in these environments. However, they do not exclude them completely. This finding coincides with that reported by Couto et al. (2015), who generally found that the benthic distribution of flora and fauna of the shallow hydrothermal system in the Azores islands was similar to that found in areas far from this hydrothermal system. Additionally, Marques-Mendes (2008) reported that the structure of the benthic community found in the hydrothermal system of the Azores islands is very similar to the community structures found in areas without hydrothermal influence. While Melwani and Kim (2008) reported that the abundance of infauna present in regions with hydrothermal influence is lower than the abundance present in areas without hydrothermal influence and where the main factor for this stratification is the high temperature present, in the hydrothermal vents of Bahía Concepción, Baja California, Mexico (78.1°C), this last factor coinciding with the work of Rodríguez-Uribe et al. (2023).

It is also highlighted that the S2 presented a similar amount of cumaceans in the three sampled zones (Z1 = 6, Z2 = 9, Z3 = 7). This could be attributed to the position of the collected organisms, which were on the sediment or partially buried without being exposed to an immediate hydrothermal discharge at the time of collection. Some researchers have described that cumaceans prefer this semi-buried position in the sediment (Heard et al. 2007, Jarquín-González and García-Madrigal 2013). Specifically, a preference for sandy sediments has been observed (Heard et al. 2007, Jarquín-González and García-Madrigal 2013, Uhlir et al. 2021). The sediments in the SWHV of Punta Mita are mostly fine and sandy in texture and well-sorted, although some mounds contain grains ranging from very fine sand to very fine gravel and even marine debris (Rodríguez-Uribe et al. 2020). Furthermore, it is important to note that there are no records available on the frequency of hydrothermal discharges at SWHV of Punta Mita; however, based on our experiences at this study site, we can suggest that several hours may pass between each hydrothermal discharge.

The global faunal composition of cumaceans associated with shallow-water hydrothermal vents is mostly represented by members of the Bodotriidae and Diastylidae families (Table 3), which was also observed in this work (Table 2); however, according to Gerken et al. (2022), some families (e.g. Ceratocumatidae, Gynodiastylidae) can be challenging to obtain despite significant collection efforts. Therefore, the presence of additional families that could increase the number of species and genera in these systems cannot be ruled out. Regarding the Mexican Pacific, Melwani and Kim (2008) reported three families (Bodotriidae, Nannastacidae and Leuconidae), three genera (*Cyclaspis*, *Cumella* and *Leucon*), one species (Cumella (Cumella) californica) and three morphospecies (*Cyclaspis* cf. sp. B, *Cumella* sp. and *Leucon* sp.) for the SWHV of Bahía Concepción, Baja California. While for the SWHV of Punta Mita, three families were also recognised (Bodotriidae, Diastylidae and Pseudocumatidae); however, five genera (*Cyclaspis*, *Diastylis*, *Oxyurostylis*, *Pseudoleptocuma* and *Pseudocuma*) and six morphospecies (*Cyclaspis* sp. 1, *Cyclaspis* sp. 2, *Diastylis* sp. 1, *Oxyurostylis* sp. 1, *Pseudoleptocuma* sp. 1 and *Pseudocuma* sp. 1) were identified. This difference in faunal composition is probably because in two (chimney and transition) of the three areas sampled by Melwani and Kim (2008), temperature values were above 50°C (Z1 = 72.5 ± 5.60; Z2 = 49.0 ± 2.88), while in the present work, the temperature values were lower at least for Z2 zones (28.32 ± 2.18); therefore, these temperature values, closer to normal environmental values (26°C), could be favouring the presence of cumaceans in the SWHV of Punta Mita.

On the other hand, of the six morphospecies recognised for the SWHV of Punta Mita, *Pseudoleptocuma* sp. 1 and *Pseudocuma* sp. 1 could potentially be considered as new species because it is the first time that these genera are recognised within the Eastern Tropical Pacific and the Mexican Pacific, respectively. The above can be supported because, like other peracarids (e.g. Tanaidacea), cumaceans lack obligate dispersive stages or phases during their life cycle, so that populations, by remaining geographically isolated, promote allopatric speciation, as well as high regional diversity, making cosmopolitan or very wide distributions questionable or unfeasible for these groups (Blazewicz-Paszkowycz et al. 2012). Furthermore, it is important to mention that, although some efforts have been made to publicise the diversity of cumaceans associated with SWHV worldwide, the identification at a specific level of five morphospecies is still pending (Table 3). The same occurs in the Mexican Pacific where, in general, 21 morphospecies remain undefined.

The above reflects the taxonomic complexity of the group and the need to use specific collection methods that guarantee representative diversity and abundance, as well as obtaining complete organisms or those with the least possible damage. Gerken (2016) mentions that, for benthic sampling, corers may not be a particularly successful technique to obtain a wide diversity of species or a large number of specimens for two reasons: the first is that, although corers can yield a large number of individuals, they generally belong to a single species; and the second is that the distribution of cumaceans frequently shows an irregular or dispersed distribution; therefore, the author suggests using other more successful techniques such as elutriation to obtain a better representation of species and even different life stages. Furthermore, due to the size of the cumaceans (1-30 mm), it is necessary to have adequate microscopic equipment in the laboratory, as well as specialist personnel or those familiar with the morphology of the group and with the necessary traditional taxonomic treatment (e.g. dissecting morphological structures, making scientific illustrations) to make appropriate descriptions and comparisons. Therefore, to advance in the ecological knowledge of the cumaceans present in the SWHV of the Mexican Pacific, it is necessary first of all to carry out continuous monitoring to collect more specimens in good condition, train new generations of taxonomists, as well as to keep the regional taxonomic inventories updated, since in this way, it will be possible to clarify the taxonomic status of the registered morphospecies, which in their great majority have been without determin their specific level for more than ten years.

Although high temperatures and low pH levels associated with hydrothermal activity in the SWHV of Punta Mita decrease the presence of cumaceans in the zones of direct influence, these organisms are not completely absent at all sampled sites. This study contributes to the knowledge of the biodiversity of the order Cumacea in extreme environments such as the SWHV since we are reporting for the first time the presence of five genera of cumaceans (*Cyclaspis*, *Pseudoleptocuma*, *Diastylis*, *Oxyurostylis* and *Pseudocuma*) in the SWHV of Punta Mita, of which two (*Pseudocuma* and *Pseudoleptocuma*) are the first records for the Mexican Pacific. Given the importance of cumaceans as indicators of sedimentary conditions and their potential for biomonitoring of marine benthos, it is suggested to continue documenting species through biological inventories, clarify the taxonomic identifications of morphospecies, as well as to include a molecular approach to establish a generic database that provides a deeper understanding of the biodiversity, ecological role, habitats, population structure, distribution and genetic pool of this group in the studied region.

## Supplementary Material

XML Treatment for Cyclaspis sp. 1

XML Treatment for Cyclaspis sp. 2

XML Treatment for Pseudoleptocuma sp. 1

XML Treatment for Diastylis sp. 1

XML Treatment for Oxyurostylis sp. 1

XML Treatment for Pseudocuma sp. 1

## Figures and Tables

**Table 1.** Total number of cumaceans and morphospecies collected at each study site of the shallow-water hydrothermal vents of Punta Mita.

Morphospecies		S1			S2			S3		
	Z1	Z2	Z3	Z1	Z2	Z3	Z1	Z2	Z3	Total
*Cyclaspis* sp. 1	0	0	0	0	0	0	0	0	2	2
*Cyclaspis* sp. 2	0	0	0	1	4	3	1	1	0	10
*Pseudoleptocuma* sp. 1	0	2	0	0	0	0	0	0	0	2
*Diastylis* sp. 1	0	0	0	0	0	1	0	0	0	1
*Oxyurostylis* sp. 1	0	0	0	2	3	3	0	0	0	8
*Pseudocuma* sp. 1	0	0	0	3	0	0	0	0	0	3
Indeterminate	0	0	0	0	2	0	0	0	1	3
Total	0	2	0	6	9	7	1	1	3	29
S1, site 1; S2, site 2; S3, site 3; Z1, zone 1; Z2, zone 2; Z3, zone 3.										

**Table 2.** Updated list of species and morphospecies of cumaceans recorded in the Mexican Pacific (own elaboration).

**Taxonomic name (Author, Year)**	**According to (Source)**
**Bodotriidae T. Scott, 1901** *Cyclaspisgiveni* Donath-Hernández, 2011 *Cyclaspisnubila* Zimmer, 1936 *Cyclaspisbituberculata* Donath-Hernández, 1988 *Cyclaspisconcepcionensis* Donath-Hernández, 1988 *Cyclaspisboquillensis* Jarquín-González & García-Madrigal, 2013 *Cyclaspishyalinus* Jarquín-González & García-Madrigal, 2013 *Cyclaspis* sp. *Cyclaspis* sp. A *Leptocumaforsmani* Zimmer, 1943	Donath-Hernández (1987), Donath-Hernández (1988), Donath-Hernández (1993), Donath-Hernández (2011a), Torres-Alfaro et al. (2012) and Jarquín-González and García-Madrigal (2013)
**Diastylidae Bate, 1856** *Anchicolurusoccidentalis* (Calman, 1912) *Diastylopsistenuis* Zimmer, 1936 *Diastyliscalderoni* Donath-Hernández, 1988 *Oxyurostylispacifica* Zimmer, 1936 *Oxyurostylistertia* Zimmer, 1943 *Oxyurostylis* sp.	Donath-Hernández (1987), Donath-Hernández (1988), Donath-Hernández (1993)and Donath-Hernández (2011a)
**Lampropidae Sars, 1878** *Alampropscarinatus* (Hart, 1930)* Alampropsquadriplicatus (Smith, 1879)** Hemilampropscalifornicus Zimmer, 1936	Donath-Hernández (1987) and Donath-Hernández (2011a)
**Nannastacidae Bate, 1866** *Campylaspis* C *Campylaspisrubromaculata* Lie, 1969 *Campylaspis* sp. A *Campylaspis* sp. B *Campylaspisbiplicata* Watling & McCann, 1996 *Cumella* sp. *Cumella* sp. A *Cumella* sp. C Cumella (Cumewingia) quintinensis Donath-Hernández, 2011 Cumella (Cumewingia) carmeinae Jarquín-González & García-Madrigal, 2013 *Elassocumellakrakeri* Jarquín-González & García-Madrigal, 2013 *Nannastacuscorallinus* Jarquín-González & García-Madrigal, 2013	Donath-Hernández (1987), Donath-Hernández (1993), Donath-Hernández (2011a), Donath-Hernández (2011b), Jarquín-González and García-Madrigal (2013) and Torres-Alfaro et al. (2012)
**Leuconidae Sars, 1878** *Coricumazurai* Jarquín-González & García-Madrigal, 2013	Jarquín-González and García-Madrigal (2013)
* Recorded as *Lampropscarinata* by Donath-Hernández (1987). ** Recorded as *Lampropscuadriplicata* by Donath-Hernández (1987).	

**Table 3.** Species and morphospecies of cumaceans in shallow-water hydrothermal vents (SWHV) and surrounding seep areas worldwide (own elaboration).

**Taxonomic name (Author, Year)**	**Location**	**Depth (m)**	**According to (Source)**
**Bodotriidae T. Scott, 1901** *Eocumaferox* (Fischer, 1872) *Iphinoeserrata* Norman, 1867	Paleohori Bay, Milos, Greece	0-10	Dando et al. (1995)
Pseudocumatidae Sars, 1878 Pseudocuma (Pseudocuma) longicorne (Bate, 1858)			
**Diastylidae Bate, 1856** *Diastylisquadriplicata* Watling & McCann, 1996 *Diastylissantamariensis* Watling & McCann, 1996 *Diastylopsisdawsoni* Smith, 1880	Northern California shelf, USA	31-53	Levin et al. (2000)
**Lampropidae Sars, 1878** *Mesolampropsdillonensis* Gladfelter, 1975			
**Leuconidae Sars, 1878** *Eudorellapacifica* Hart, 1930			
**Diastylidae Bate, 1856** *Anchicolurusoccidentalis* (Calman, 1912)	White Point, California, USA	8	Melwani and Kim (2008)
**Lampropidae Sars, 1878** *Lamprops* sp.			
**Bodotridae T. Scott, 1901** *Cyclaspis* cf. sp. B	Bahía Concepción, Mexico	12	Melwani and Kim (2008)
**Nannastacidae Bate, 1866** Cumella (Cumella) californica Watling & McCann, 1996 *Cumella* sp.			
**Leuconidae Sars, 1878** *Leucon* sp.			
Cumacea sp. A	Tutum Bay, Ambitle Island, Papua New Guinea	60	Karlen et al. (2010)
